# Do Cortisol and Dehydroepiandrosterone Influence Motivational Factors for Non-Suicidal Self-Injury in Female Adolescents?

**DOI:** 10.3390/jcm12051924

**Published:** 2023-02-28

**Authors:** Francesco Maria Piarulli, Anna Margari, Francesco Margari, Emilia Matera, Federica Croce, Flora Furente, Alessandra Gabellone, Maria Giuseppina Petruzzelli

**Affiliations:** 1 Department of Translational Biomedicine and Neurosciences (DiBraiN), University “A. Moro”, 7016 Bari, Italy; 2 Department of Precision and Regenerative Medicine and Ionian Area (DiMePRe-J), University “A. Moro”, 7016 Bari, Italy

**Keywords:** non-suicidal self-injury, stress response, cortisol, DHEA-S, emotional dysregulation, theory of urgency, Ottawa Self Injury Inventory

## Abstract

Non-suicidal self-injury (NSSI) is a significant public health issue that particularly affects female adolescents usually emerging during puberty, with a subsequent reduction and even remission in the phenomenon later in life. The dysregulation of the hormonal stress response, particularly cortisol and dehydroepiandrosterone sulfate (DHEA-S), whose levels increase markedly during pubertal adrenarche, has been associated with the development and maintenance of a wide range of emotional disorders. Our study aims to investigate whether different cortisol-DHEA-S response patterns could be associated with the main motivational moderators to engage NSSI as well as with urgency and motivation to stop NSSI in a sample of female adolescents. We found significant correlations between stress hormones and several factors that support and sustain NSSI, specifically: cortisol levels and distressing/upsetting urge (r = 0.39 and a *p* = 8.94 × 10^−3^) and sensation seeking (r = −0.32 and a *p* = 0.04), as well as cortisol/DHEA-s ratio and external emotion regulation (r = 0.40 and a *p* = 0.01) and desire to stop NSSI (r = 0.40 and a *p* = 0.01). Cortisol and DHEA-S may play a role in NSSI through the regulation of stress responses and affective states. Such results could have implications for the development of new and improved treatment and prevention plans for NSSI.

## 1. Introduction

Non-suicidal self-injury (NSSI) is defined as the voluntary self-infliction of damage to one’s own body surface in a manner that causes bleeding, bruising, or pain without the expectation to produce serious and life-threatening harm [1,2,3]. The prevalence of NSSI ranges from 13 to 28% in community-based samples [4] and up to 80% in inpatient settings [5]. It usually onsets during puberty and develops throughout adolescent age, plateauing in early adulthood and most commonly affecting females [2,6]. Although subjects who engage in NSSI do not have a conscious suicidal intent, NSSI is considered a risk factor for suicide attempts and is strongly associated with other risk behaviors and comorbid psychopathology [7,8,9,10,11,12,13,14,15]. Numerous previous studies have argued in favor of the need of separating the clinical entity of NSSI from other entities, specifically from suicidal behavior: it is indeed likely that the two are underlined by different risk factors, clinical profiles both in psychopathology, treatment, and prevention, with NSSI lacking suicidal ideation and providing a dysfunctional relief mechanism from stressful feelings and negative emotions [16,17]. In line with the evidence from such studies, in the recently published fifth edition of the Diagnostic and Statistical Manual of Mental Disorders Fifth Edition, Text Revision (DSM-5-TR), diagnostic codes for suicidal behavior and non-suicidal self-injury were added (Section 2, chapter “Other conditions that may be a focus of clinical attention”) to help clinicians better document these behaviors when they occur with other mental illnesses [18].

Evidence suggests that a complex interaction of psychosocial and biological factors may contribute to the risk of self-injurious behavior. Although genetic predisposition plays an important role, it does not appear to play a major role and is consistent with a variety of conditions including ADHD, depression, income, anorexia, neuroticism, and suicidal ideation [19,20]. Recent focus has been put on the impact of psychoneuroendocrinology, with increasing evidence for the role of the hypothalamic–pituitary–adrenal (HPA) axis, which is, among other functions, responsible for the hormonal response to stressful factors [21]. Genetic, neuroendocrine, and stressful environmental factors likely interact to increase the risk for emotional dysregulation and NSSI [21,22]. A feature of emotional dysregulation is common in people who trigger NSSI, leading to difficulties in the ability to be aware of, understand, and accept one’s emotions, inhibit impulsive behaviors related to emotional distress, and avoid activities that may trigger negative emotions. The observation of social patterns, self-punishment, communication of high-intensity behaviors, and implicit identification with self-injurious behaviors may explain why some people use NSSI to regulate their negative emotional/cognitive experiences [23]. Moreover, according to the Theory of Urgency [24], some people are more prone to impulsive and potentially harmful behaviors such as NSSI when they experience a negative affective state. According to this theory, the mechanism of Urgency involved in NSSI might be enhanced not only by reducing negative emotions but also by generating positive ones [25,26].

Although genetics and temperament may partly account for problematic behavior, physiological activity in general and the way adolescents respond physiologically to stressors may also contribute. Excessive or insufficient physiological activity may underlie externalizing and internalizing behaviors, such that youths engage in certain behaviors to modulate internal physiological cues (e.g., engaging in aggressive behaviors to stimulate increased physiological activity or withdrawing from social situations because of heightened physiological stimulation) [27,28]. The HPA axis plays a critical role in the body’s adaptive stress response, particularly when perceived or actual threats have a social-evaluative component (e.g., peer rejection or classroom presentations) or are uncontrollable (e.g., being in a chaotic home environment). The HPA axis represents a neuroendocrine cascade that connects the hypothalamus to the pituitary and adrenal glands [29]. Cortisol and dehydroepiandrosterone (DHEA) are the most abundant circulating hormones of the entire HPA axis [30] and exert pleiotropic physiological effects, including the regulation of immune, metabolic, and cognitive functions. Cortisol supports acute stress responses by drawing on the energy reserves of the brain and body, i.e., increasing the catabolic state of the body [31]. DHEA, which circulates in the blood primarily as dehydroepiandrosterone sulfate (DHEA-S), has anti-glucocorticoid effects that can occur through a variety of mechanisms, including indirectly enhancing the body’s anabolic state, and experiences an increase in synthesis during puberty, following adrenarche [32]. Cortisol and DHEA are thought to serve opposite functions, with DHEA attenuating the effects of cortisol [33]. Given the reciprocal and often opposing effects of cortisol and DHEA-S, the use of a cortisol/DHEA-S ratio may provide an overall measure of the stress response hormone profile and a stable indicator for daily and age-related changes in these hormone levels [34]. For instance, previous studies found high cortisol and low DHEA-S levels in depressed and anxious adolescents, and higher DHEA and lower cortisol levels correlated with lower severity of externalizing disorders such as oppositional defiant behavior and conduct disorder and lower severity of ADHD [35,36,37].

Previous literature investigating the relationship between stress hormones and suicidal behavior often found conflicting results, showing higher cortisol levels in subjects with suicidal behavior compared to healthy controls but lower cortisol levels compared to subjects diagnosed with other psychiatric diagnoses without suicidal behavior [38,39].

Given the reported differences between suicidal behavior and NSSI, it is likely to hypothesize a specific stress-hormone pattern regarding NSSI; however, there are currently limited and conflicting data on the possible relationship between NSSI and stress hormones or changes in HPA axis functionality [40]. Considering that NSSI is associated with impairments in stress regulation and emotion recognition and is more common in females during the post-adrenarche period, we hypothesized that different cortisol–DHEA-S responsivity patterns might be associated with the principal motivational moderators in female adolescents who engage in NSSI. Therefore, we selected a sample of female adolescents with a history of NSSI and examined both NSSI functions and circulating levels of stress hormones related to the HPA axis. The motivational moderators of NSSI were examined using the OSI with specific regard to the four motivational functions (Internal Emotion Regulation, IER, External Emotion Regulation, EER, Social Influence, SI, Sensation Seeking, SS), the type of urgency preceding self-harm (distressing/upsetting or comforting or intrusive/invasive), and the level of motivation to stop self-injury (not at all, somewhat, or extremely motivated). Circulating cortisol and DHEA-S levels as well as the cortisol/DHEA-S ratio were assessed as indicative of the neuroendocrine response to stress. The main exploratory aim of this study was to verify whether there was a significant correlation between cortisol, DHEA-S, and the cortisol/DHEA-S ratio with the four motivational functions and the type of urge or motivation to stop NSSI, respectively.

## 2. Materials and Methods

### 2.1. Subjects

We enrolled adolescent patients referred to the Operative Units of Psychiatry and Child Neuropsychiatry at the University Hospital of Bari, Italy, for various psychiatric disorders between January 2018 and April 2022. We included patients with current NSSI who were screened using DSM-5 criteria for “Nonsuicidal self-injury” [18,41] with various neuropsychiatric diagnoses. Exclusion criteria included the presence of medical conditions that precluded active collaboration during the assessment, including intellectual disability. Psychiatric diagnoses were performed, according to DSM-5 criteria by an experienced psychiatrist/child neuropsychiatrist based on clinical symptoms and medical history collection. The study was conducted according to the guidelines of the Declaration of Helsinki and approved by the Ethics Committee of the Azienda Ospedaliero Universitaria Consorziale Policlinico of Bari (resolution no. 1761, December 2019).

### 2.2. Clinical Assessment

Psychiatric diagnoses were performed, according to DSM-5 criteria, by an experienced psychiatrist/child neuropsychiatrist based on clinical symptoms and medical history collection.

All participants underwent the OSI [42,43], a 27-item self-administered questionnaire that fully assesses and examines the cognitive, affective, behavioral, motivational, and environmental aspects of the NSSI. The results from OSI include quantitative raw scores on specific aspects of self-injury (e.g., frequency of and thoughts about it, type of injury, the usefulness of any previous treatments, etc.) and complex qualitative mean motivational items that categorize the main putative motivational factors behind NSSI. The test does not provide total and cut-off scores because the items include both quantitative and qualitative responses. For this reason, our study used a comprehensive approach that employed both quantitative and qualitative measures. The OSI has been shown to be valid and reliable in a university sample of young adults, with internal consistency values ranging from 0.67 to 0.87, and is suitable for use in clinical samples of adolescents. An adapted OSI Italian version from Nixon et al. [43] was used. To achieve the objectives of our study, we used the following items:

- No. 9 (“When you get the urge to hurt yourself: The urge is distressing/upsetting; The urge is comforting; The urge is intrusive/invasive”), where for each of the three possible urges for each subject the scores range from 0 (not at all) to 4 (extremely);
- No. 14 (“Why do you think you started and if you continue, why do you still self-injure without meaning to kill yourself?”), where the scores range from 0 (never a reason) to 4 (always a reason), and are subsequently scored in terms of IER (the total score, variable between 0 and 24, is the sum of the scores of subitems 4, 6, 9, 14, 16, 18), SI (the total score, variable between 0 and 28, is the sum of the scores of subitems 3, 9, 10, 11, 13, 15, 21), EER (the total score, variable between 0 and 12, is the sum of the scores of subitems 1, 12, 20), SS (the total score, variable between 0 and 16, is the sum of the scores of subitems 2, 7, 22, 23);
- No. 22 (“How motivated are you at this time to stop self-injuring?”), where scores range from 0 (not motivated at all) to 4 (extremely motivated).

### 2.3. Laboratory Analysis

Serum levels of cortisol and DHEA-s were measured at 8:00 a.m. during inpatient hospitalization by immunofluorescence assay of blood samples. The blood sample was collected by an experienced nurse and analyzed by the local clinical pathology laboratory. Raw values and mean reference values from our laboratory were collected (serum cortisol: 48–195 μgL; serum DHEA-s: 24–368 μg/dL). The cortisol/DHEA-S ratio was calculated from the raw values.

### 2.4. Statistical Analysis

We performed a descriptive analysis of frequency, mean, standard deviation (SD), range, and median values for the clinical and hormonal data. A Pearson correlation test was used to test whether there were correlations between serum levels of cortisol, DHEA-S, and the cortisol/DHEA-s ratio and the mean scores of items 9, 14, and 22 of the OSI. R software for MacOS was used to process the data. The significance level was set at a *p*-value of >0.05.

## 3. Results

The total sample included 43 female adolescents with different psychiatric diagnoses. The main clinical diagnoses according to DSM-5 are listed in Table 1. The mean age was 15.23 years (SD ± 1.74). All patients were post-pubescent and had already presented the menarche.

Circulating levels of hormones for the sample are shown in Table 2.

The mean values and SD of the OSI 9, 14, and 22 items are shown in Table 3.

A Pearson correlation between cortisol and the Urge distressing/upsetting score was significant with a Pearson’s r = 0.39 and a *p* = 8.94 × 10^−3^. We found no other significant correlations between hormones and other types of urges.

A Pearson correlation between the cortisol/DHEA-S ratio and the EER mean score of item 14 was significant with a Pearson’s r = −0.37 and a *p* = 0.02. A Pearson correlation between cortisol and the SS mean score of item 14 was significant with a Pearson’s r = −0.32 and a *p* = 0.04. We found no other significant correlations between hormones and other types of mean scores derived from item 14.

A Pearson correlation between the cortisol/DHEA-S ratio and OSI item 22 “Motivation to stop NSSI” was significant with a Pearson’s r = 0.40 and a *p* = 0.01.

**Figure 1:**
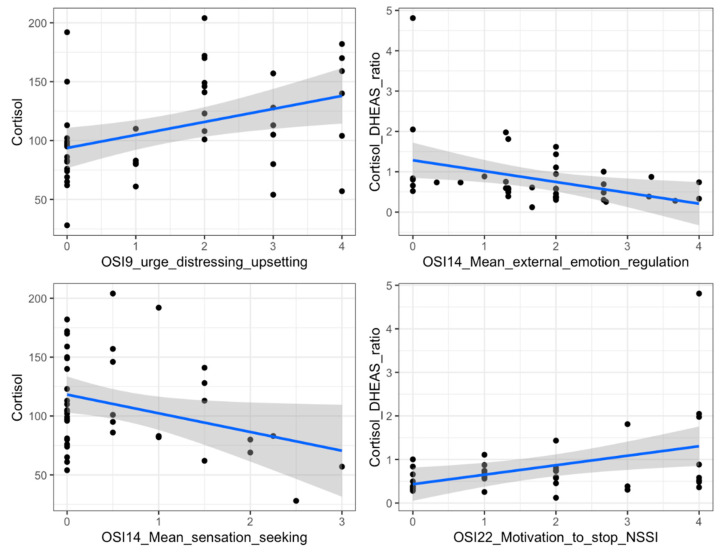
Correlation scatter plots: in the (**upper left**) plot, the correlation between cortisol and OSI no. 9 (“Urge”) item score “Urge distressing/upsetting”; in the (**upper right**) plot, the correlation between the cortisol/DHEA-S ratio and OSI no. 14 item score “Mean external emotion regulation”; in the (**lower left**) plot, the correlation between cortisol and OSI no. 14 (“Mean sensation seeking”); in the (**lower right**) plot, the correlation between cortisol/DHEA-S ratio and OSI no. 22 item score “Motivation to stop NSSI”.

Scatter plots for significant correlations are shown in Figure 1.

## 4. Discussion

In this study, we investigated the relationship between adrenocortical stress hormones and the main motivational components of self-injurious behavior in a sample of adolescent female patients with various psychiatric disorders coexisting with current NSSI. The significant results we found were a direct correlation between the cortisol level and the “urge is distressing/upsetting” option of the OSI item 9, an inverse correlation between the cortisol level and SS as well as between the cortisol/DHEA-S ratio and EER of the OSI item 14, and a direct correlation between the cortisol/DHEA-S ratio and the motivation to stop self-injuring of the OSI item 22.

Our sample consisted of adolescent female patients in whom NSSI coexisted particularly with depressive, anxiety, and personality disorders, so the data from this study are consistent with most of the available literature [3,4,5]. The decision not to include male patients was due to previously reported sex differences in adrenocortical hormone levels between males and females both in healthy subjects and in NSSI patients [44], so it would have been inaccurate and non-homogeneous to evaluate a two-sex NSSI group and study the effects of adrenocortical hormones. Furthermore, NSSI itself shows great differences between the sexes, with female adolescents being more frequently affected and having a more homogeneous clinical profile in terms of the type of injury [45]. The association between NSSI, adolescence, and the female gender is consistent with the importance of the role that biological hormonal components play. Adolescence is a transitional phase defined as the period between the onset of puberty and the beginning of self-sufficiency and is characterized by the continuous structural maturation of the brain and hormonal changes beginning at puberty [46].

Sex differences in the nature and timing of puberty may have differential effects on brain development, affecting behavior and persisting into adulthood. In females, the gonadarchic pubertal process, the phase of puberty characterized by sex-specific increases in estrogen and testosterone levels, begins one to two years earlier than in males [47,48,49]. This period coincides with the onset of HPA axis reactivity to psychological stress, sex differences in negative affect, subjective experience, and the behavioral expression of negative emotions in response to stressful situations [50]. In view of this, both sex hormones and the timing of puberty may be involved in the pathophysiology of self-injurious behavior and the development of maladaptive coping strategies through the modulation of the neuroendocrine system, with the consequence of presenting NSSI with differences in clinical symptomatology, gravity, and types of lesions between males and females [22]. Furthermore, stress-related disorders-not only NSSI but also the various associated disorders such as depression, anxiety, and PTSD-are known to be associated with dysfunction of the HPA axis and prefrontal cortex, suggesting a functional link between aberrant prefrontal corticosteroid signaling, stress adaptation, emotional control, mood regulation, and the pathway by which stress can lead to psychopathology [51].

The first significant finding of our study was the direct correlation between cortisol levels and the “The urge is distressing/upsetting” score of item no. 9 of the OSI. There are no previous findings on this topic among individuals with NSSI. The authors who examined the relationship between negative urgency and alcohol seeking found that negative urgency was not so much related to the release of cortisol as it was to the induction of negative mood conditions, suggesting that the context of mood should be considered when measuring behavioral indicators of negative urges. Consequently, the negative mood becomes a risk factor only for those who exhibit higher levels of negative urgency [52]. There is ample evidence that stress hormone release is associated with an increase in negative affective states that cause stress, whereas positive affective states are associated with a decrease in cortisol concentration. The urge to NSSI, the strong desire to engage in self-injurious behavior, has been described as one of the precursors of this behavior, both in solitary and socializing activities [23,53]. More specifically, NSSI urges have been found to be positively associated with the occurrence and intensity of negative affective states, which are certainly a cause of interpersonal and intrapersonal discomfort (hostility, guilt, sadness, anger, and rejection) [53,54,55]. This is consistent with the four-function model of NSSI [56], which states that some individuals engage in NSSI to alleviate negative intrapersonal or interpersonal experiences [6,40,57,58].

The second significant finding of our study was the inverse correlation between cortisol level and SS and cortisol/DHEA-s ratio and EER of item 14 of OSI.

Pharmacological studies in healthy subjects have shown that cortisol administration before a stressful event leads to an attenuation of negative affect [59,60,61,62], supporting a protective role of cortisol in coping with the emotional burden of a stressful situation [60]. Although the short-term activation of the HPA axis has an adaptive function, prolonged and/or repeated stress leads to the dysregulation of this system, contributing to an increased risk of disease [63], especially in young people suffering from chronic stress. According to a study by Kliewer [64], the negative association between a stressful situation and cortisol production could reflect the downregulation of the HPA axis to minimize the physiological damage caused by repetitive arousal of the stress response system, which may increase the vulnerability of young people to mental and physical health problems. Previous results have pointed to sex differences in the processing of emotional material [65,66,67] and have shown that females exhibit greater physiological and neuropsychological reactivity to emotional material compared to males because cortisol reduces negative affect in response to adverse situations following stress [59,60,61]. Research on social cognition supports the idea that this effect in women may be mediated by oxytocin [68], a hormone of the posterior pituitary gland that is associated with parasympathetic activation and may play a counterregulatory role in psychological stress responses [69]. Although oxytocin is present in both males and females, testosterone is known to suppress the activities of oxytocin and the oxytocin receptor [69]. The stress response is traditionally characterized as “fight or flight,” in which elevated cortisol levels and enhanced peripheral blood flow support muscle movements in support of aggressive behavior or flight. Although this may promote male survival, a pivotal study by Taylor and colleagues has shown that fight or flight is not the adaptive response of females seeking to protect their offspring from threats. Oxytocin, which has anxiolytic, anti-inflammatory, analgesic, and healing effects, generally provides evolutionary benefits in pregnancy, labor, delivery, breastfeeding/caregiving, and social bonding. Lastly, it improves cognitive performance in stressful situations, whereas cortisol mobilizes the metabolic resources needed for rapid decision-making [50]. Cognitive control involves the ability to focus on the most important aspects of a situation and to direct attention to the behaviors needed to achieve the desired goal. It is therefore possible that higher levels of oxytocin in women are associated with better cognitive-emotional accuracy in the context of stress. Oxytocin could play a role in helping women process emotional information accurately and maintain a more positive attitude during stress [70,71]. Based on these assumptions, it is possible to deduce that there is an association between cognitive and emotional self-regulation thanks to common mental control mechanisms [72]. Although several studies suggest a link between hormones and emotion regulation, different hormones seem to exert their influence differently depending on the emotional stress context and on the emotion regulation strategies activated. These data could explain why the significant correlations obtained in our study involve only two types of NSSI functions and why specifically in the context of social influence and external emotion-regulating factors that mediate NSSI. Furthermore, a recent meta-analysis suggests that hormonal responsiveness to emotional situations in people with mental disorders differs from that of healthy individuals; according to this analysis, cortisol responsiveness to stressors is attenuated in women with affective disorders such as anxiety and depression [73].

The last significant finding of this study was a direct correlation between the cortisol/DHEA-S ratio and motivation to stop self-injuring of item no. 22 of the OSI. This result is consistent with our previous finding regarding the positive correlation between cortisol levels and Urge distressing/upsetting: it is possible that the increase in cortisol, linked to a negative emotional state associated with a feeling of urgency that causes discomfort, may affect greater motivation to stop self-harming. A previous study by Turner et al. created a specific scale to assess the specific reasons for stopping NSSI, highlighting the importance of nine individual factors that correlate with the intention to stop self-harming. The nine factors could be divided into two groups: a first, conceptualized as vulnerability-related reasons (namely, Fear of Discovery and Stigma, concern regarding Negative Physical Consequences of NSSI, motivation due to Others’ Expectations, concern about Addiction to NSSI, and reliance on Situational and Environmental Deterrents to stop NSSI) and a second that has been conceptualized as resiliency-related reasons (namely, Desire for Change and Resolution of Distress, concern regarding Negative Emotional Consequences of NSSI, Body Concerns, and concern about NSSI’s Negative Impact on Relationships) [74]. Given the non-specificity of the single data on the desire to stop analyzed in our study and based on our other findings, we argue for a dual effect mediated by cortisol and DHEA-S on vulnerability-related reasons: the increase in cortisol, directly or via the mediating of the increase in urge, could enhance a vulnerability-related desire to stop. On the other hand, a decrease in DHEA-s could hinder resiliency [75,76], furthering vulnerability-related reasons to stop.

## 5. Conclusions

There is a probable association between the hormonal mechanisms underlying stress responses and the motivations that maintain self-injurious behavior, particularly the need for distress, emotional regulation, sensation seeking, and the desire to stop self-injurious behavior. For a better understanding of the neurobiology of non-suicidal self-injury, longitudinal studies are needed to help distinguish between predisposing biological anomalies and the biological consequences of self-harm. The growing recognition of the role of stress hormones in the ANS raises questions about treatment options targeting stressors, as well as the utility of psychotherapeutic techniques aimed at improving the psychological management of stressful situations and potential prevention programs. Previous studies have shown the potential of integrating psychoeducation about NSSI in evidence-based school interventions aimed at preventing mental conditions. Such prevention programs were well received by students and school staff, increased awareness of self-harm, and improved help-seeking attitudes skills among students [77,78,79]. In light of our results, it is important that psychoeducation focuses on the importance of stressful factors, altered stress response, and emotional dysregulation as early indicators of distress.

Future studies could evaluate the data we studied in a separate cohort composed of male NSSI patients in order to evaluate different stress hormonal profiles related to specific motivations for self-injury. Given the important gender-mediated differences already pointed out, other hormonal stress response profiles could be more specific for the male gender. In this line, expanding the palette of hormones to include other HPA hormones could elucidate other mechanisms underlying stress, gender, and NSSI. In addition, a different case–control approach using matched non-self-injury subjects comparing levels and trajectories of adrenocortical hormones could be useful to better elucidate the way in which stress response is involved in causing and sustaining NSSI.

## 6. Limitations

The limitations of this study included the small sample size, the lack of males, and matched non-self-injury subjects. Our sample includes several diagnoses known to correlate with an increase or decrease in stress hormones, such as anxiety and depression. Normative levels of cortisol are elevated and increase by 50–70% to their peak during the first 30 to 40 min after awakening. Our cortisol data, representing morning cortisol blood levels, may be influenced by this physiological phenomenon.

## Figures and Tables

**Table 1:** Diagnostic and Statistical Manual of Mental Disorders Fifth Edition (DSM-5) diagnosis of the sample.

DSM-5 Diagnosis	Frequency	Percent
Depressive disorders	30	69.77
Anxiety disorders	15	34.88
Personality disorders	15	34.88
Neurodevelopmental disorders	14	32.56
Nutrition and eating disorders	11	25.58
Substance-related and addictive disorders	10	23.26
Bipolar disorders	6	13.95
Traumatic and stressful events related disorders	4	9.30
Schizophrenia spectrum disorders	3	6.98
Disruptive behavior, impulse control, and conduct disorders	3	6.98
Somatic symptoms and related disorders	2	4.65

**Table 2:** Circulating levels of hormones for the sample.

	Mean	SD	Range	Minimum	Maximum
Cortisol (μg/L)	110.40	41.71	176.00	28.00	204.00
dehydroepiandrosterone sulfate DHEA-s (μg/dL)	176.01	78.26	430.20	15.80	446.00
Cortisol/DHEA-s ratio	0.86	0.82	4.69	0.12	4.81

**Table 3:** Mean values and SD of the Ottawa Self Injury Inventory, items 9, 14, and 22.

	Mean	SD	Range	Minimum	Maximum
OSI9_urge_distressing_upsetting	1.51	1.49	4.00	0.00	4.00
OSI9_urge_conforting	1.81	1.42	4.00	0.00	4.00
OSI9_urge_intrusive_invasive	1.98	1.52	4.00	0.00	4.00
OSI14_Mean_external_emotion_regulation	1.74	1.08	4.00	0.00	4.00
OSI14_Mean_sensation_seeking	0.58	0.84	3.00	0.00	3.00
OSI22_Motivation_to_stop_NSSI	1.74	1.48	4.00	0.00	4.00

## Data Availability

No new data were created or analyzed in this study. Data sharing is not applicable to this article.
